# 

**Published:** 1867-06

**Authors:** 


### AMERICAN DENTAL ASSOCIATION.

THE American Dental Association will hold its Seventh Annual Meeting in Cincinnati, on Tuesday, July 30th, 1867. There is good reason to hope that this will be the most profitable meeting of the profession ever held. The great increase of local and State societies, the consequent increase of delegates, the large attendance of permanent members, the place of meeting being central, all indicate a good meeting. Let there be a thorough reunion of our profession, which was never separated or divided in brotherly feeling, however long they may have been kept asunder by adverse circumstances beyond their control. The profession in this vicinity are expecting their brethren and friends, regardless of distance or geography. Come on! brethren. Cincinnati is a good place to come to. If you don't believe it, ask your family physicians who have just got home from the meeting of the "American Medical Association." W.

### OHIO STATE DENTAL SOCIETY.

The Ohio State Dental Society will hold its next semi-annual meeting in Columbus on Tuesday, the 18th of June. The season of the year will be pleasant. The place is central. The few days necessary to attend the meeting could not be more profitably spent otherwise by any Dentist in the State. The constitution limits the admission of members to the regular meetings. It is hoped that many new members may be admitted at this meeting. Let us all attend. W.

### AMERICAN DENTAL ASSOCIATION.

This body meets, according to adjournment, in the City of Cincinnati, on the last Tuesday of July. It is confidently expected that there will be a large attendance; probably larger than ever before. In order that the meeting be one of interest and profit, all the members should come with the full purpose of accomplishing the most possible. Every one should come with a purpose and a preparation to do his full share of the work. The responsibility of making the meeting an interesting one does not rest upon the officers, nor upon any committee, but it does rest upon *all the members* who may be present, and hence we hope that every one will bring his contribution, and time shall be afforded for its presentation. The time of the Association, so far at least, as the arrangements of the Executive Committee are concerned, shall be devoted exclusively to its legitimate work. Outside attractions will be wholly ignored during the time of the sessions. We say this because of a thorough conviction that the members of the Association wish to have it so.

The Executive Committee have made ample arrangements for the accommodation of the Association. Hopkins' Music Hall, on Fourth Street, near Elm, a very excellent room, has been secured. There are sufficient ante rooms, and a fine room for clinics.

The Committee will be at the room as early as 8 o'clock on the morning of the first day, for the purpose of receiving the credentials of new members. It is desirable that that part of the business be done before the regular hour of meeting, so far as practicable. Delegates will therefore report early. Arrangements have been made for the accommodation of the members, with the Burnet House, the Clarendon Hotel, and the Saint James, in either of which the accommodations and arrangements will be all that the most fastidious could desire. Any parties wishing to secure rooms can do so by notifying Dr. H. R. Smith, of this city, who will give prompt attention to such requests.

Very efficient arrangements have been made for the presentation and exhibition of instruments and appliances. We hope that all members having any thing new and valuable, or even though it may be old and valuable, and not generally known to the profession, will not fail to bring it in. A fixed time each day has been assigned for such exhibition.

Any articles for exhibition consigned to Dr. H. R. Smith, will be received and taken care of by him.

J. TAFT,

W. H. GODDARD, } *Com. of Arrangements.*

H. R. SMITH,

### CONTENTS.

### The Dental Register

### OF THE WEST.

*Issued Monthly, at $3 a Year in Advance.*

DEVOTED TO THE INTERESTS OF THE DENTAL PROFESSION.

EDITED BY J. TAFT AND G. WATT.

The volume commences in January and closes in December.

The Register being issued monthly is always fresh, therefore indispensable to the practitioner who desires to keep posted up with the new inventions and improvements which are daily developed. Neither expense nor effort will be spared to give value and variety to its contents. Articles will be illustrated with well executed engravings whenever they will add to the interest or assist in explanation.

Its extensive circulation and frequency of issue makes it one of the BEST DENTAL ADVERTISING MEDIUMS in the United States.

RATES OF ADVERTISING.

One page, 1 year, $50. Half page, 1 year, $30. Quarter page, or card, 1 year $20.

All advertising bills payable in advance, or at furthest after the issue of the first number containing the advertisement. All transient advertisements to be paid for in advance.

☞ CONTRIBUTIONS SOLICITED.

Address, J. TAFT, or "DENTAL REGISTER," Cincinnati, Ohio.

Business Communications to

**J. TAFT, Publisher,**
No. 238 Fourth St., between Plum and Central Avenue.

